# Benzimidazoles Promote Anti-TNF Mediated Induction of Regulatory Macrophages and Enhance Therapeutic Efficacy in a Murine Model

**DOI:** 10.1093/ecco-jcc/jjx104

**Published:** 2017-08-16

**Authors:** Manon E Wildenberg, Alon D Levin, Alessandro Ceroni, Zhen Guo, Pim J Koelink, Theodorus B M Hakvoort, Liset Westera, Felicia M Bloemendaal, Johannan F Brandse, Alison Simmons, Geert R D’Haens, Daniel Ebner, Gijs R van den Brink

**Affiliations:** 1 Department of Gastroenterology and Hepatology, Academic Medical Center, The Netherlands; 2 Tytgat Institute for Intestinal and Liver Research, Academic Medical Center, The Netherlands; 3 Target Discovery Institute, Nuffield Department of Medicine, University of Oxford, UK; 4 Weatherall Institute of Molecular Medicine, University of Oxford, UK

**Keywords:** Anti-TNF, colitis, high throughput screen, macrophage

## Abstract

**Background and Aims:**

Regulatory macrophages play a critical role in tissue repair, and we have previously shown that anti-tumour necrosis factor [TNF] antibodies induce these macrophages *in vitro* and *in vivo* in IBD patients. The induction of regulatory macrophages can be potentiated using the combination of anti-TNF and thiopurines, consistent with the enhanced efficacy of this combination therapy described in clinical trials. As thiopurines are unfortunately associated with significant side effects, we here aimed to identify alternatives for combination therapy with anti-TNF, using the macrophage induction model as a screening tool.

**Methods:**

Mixed lymphocyte reactions were treated with anti-TNF and a library of 1600 drug compounds. Induction of CD14+CD206+ macrophages was analysed by flow cytometry. Positive hits were validated *in vitro* and in the T cell transfer model of colitis.

**Results:**

Among the 98 compounds potentiating the induction of regulatory macrophages by anti-TNF were six benzimidazoles, including albendazole. Albendazole treatment in the presence of anti-TNF resulted in alterations in the tubulin skeleton and signalling though AMPK, which was required for the enhanced induction. Combination therapy also increased expression levels of the immunoregulatory cytokine IL-10. *In vivo*, albendazole plus anti-TNF combination therapy was superior to monotherapy in a model of colitis, in terms of both induction of regulatory macrophages and improvement of clinical symptoms.

**Conclusions:**

Albendazole enhances the induction of regulatory macrophages by anti-TNF and potentiates clinical efficacy in murine colitis. Given its favourable safety profile, these data indicate that the repurposing of albendazole may be a novel option for anti-TNF combination therapy in IBD.

## 1. Introduction

One of the major therapeutic objectives in the management of inflammatory bowel disease [IBD] is mucosal healing of the intestine. Currently, anti-tumour necrosis factor [TNF] monoclonal antibodies are the most effective treatment to achieve this therapeutic goal. However, despite its obvious success, anti-TNF monotherapy does not result in complete mucosal healing in all patients. In a landmark clinical trial, complete mucosal healing was observed in 30% of patients after 26 weeks of monotherapy with infliximab [IFX].^1^ Response rates were increased to 44% by combining IFX therapy with the thiopurine azathioprine, and in most centres this combination has now become the standard of care in patients who fail conventional therapy.

Wound healing is an active process involving both immune suppression and repair of the damaged tissue. A key role in this process is played by regulatory macrophages, which inhibit the active immune response, remove apoptotic cells, and promote epithelial proliferation and wound contraction.^2^ Ablation of macrophages in the context of wounding results in significant delays in wound closure and increased inflammatory activity.^3^ We have previously shown that anti-TNF antibodies induce CD14+ CD163+ CD206+ M2-type regulatory macrophages which are highly suppressive of T lymphocyte proliferation and promote wound repair *in vitro*.^4^^,^^5^ Given the pivotal role of macrophages in wound healing, this is likely to be an important mechanism by which anti-TNF mediates mucosal healing. Indeed, we subsequently found that regulatory macrophages are induced in endoscopic responders to IFX but not in endoscopic non-responders.^4^ In line with the clinical additive effect of thiopurines and anti-TNF, we also observed that thiopurines enhance the induction of regulatory macrophages both in number and in immunosuppressive potential.

Unfortunately, thiopurines often have to be discontinued due to either intolerance or serious side effects. In one large Dutch inception cohort of 363 patients, thiopurines had to be stopped in 39% of patients due to adverse events.^6^ Given this problematic safety profile, alternative drugs for use as anti-TNF co-medication would fill a currently unmet need. Here, we used our *in vitro* regulatory macrophage induction assay as a screening tool for the identification of potential alternative candidates to increase anti-TNF induced mucosal healing.

## 2. Materials and Methods

### 2.1. Mixed lymphocyte reactions

Peripheral blood mononuclear cells [PBMC] were isolated from buffy coats obtained from healthy blood bank donors, using Ficoll density gradient centrifugation. PBMC of two individual donors were cultured in a 1:1 ratio for 48 h. Subsequently, anti-TNF [infliximab] or control IgG [Sigma Aldrich, Zwijndrecht, The Netherlands] was added, to a final concentration of 10 µg/ml. Where appropriate, additional compounds were added at the concentrations indicated. Albendazole [#A4673], mebendazole [#46404], docetaxel [#01885], paclitaxel [#T7402], budesonide [#PHR1178], fenbendazole [#35032], pyrythione zinc [#H6377], ciclopirox olamine [#C0415], pyrimethamine [#46706], quinacrine [#Q3251], mycophenolic acid [#M3536], adapalene [#A7486], amitryptiline [#A8404], duloxetine [#SML0474], triamterene [#T4143], and colchicine [#C9754] were all obtained from Sigma Aldrich. AMPK activator A769662 was obtained from SelleckChem [#S2697, Munich, Germany] and Compound C from Calbiochem [#171260–1, San Diego, CA]. Cultures were incubated for another 4–5 days and analysed by flow cytometry.

For analysis of effects on isolated macrophages, mixed lymphocyte reactions (MLR) were cultured for 48 h. CD14-expressing cells were isolated using magnetic cell sorting [CD14-microbeads, Miltenyi, Bergisch Gladbach, Germany] and cultured for an additional 72 h in the presence of anti-TNF and/or albendazole.

### 2.2. Flow cytometry

For flow cytometry of cell cultures, cells were harvested using 5 mM EDTA and stained using αCD14-PE [clone MϕP90], αCD206-APC [clone 19.2], αCD163-PE [clone GHI/61], and αCD86-FITC [clone FUN-1, all obtained from BD Biosciences, San Jose, CA], αCD14-PE–Cy7 [clone HCD14], and αHLA-DR-AF700 [clone L243, both obtained from Biolegend, San Diego, CA]. Cells were analysed using a FACS Fortessa [BD] and analysed using FlowJo software [Treestar Inc., Ashland, OR].

Tubulin polymerisation was measured as previously described.^7^ Briefly, KG1 or THP1 cells were incubated with compounds as indicated for 18 h, followed by fixation in warm 0.5% glutaraldehyde in microtubule stabilisation buffer [80 mM Pipes, pH 6.8, 1 mM MgCl_2_, 5 mM EDTA, and 0.5% Triton X-100]. Subsequently samples were quenched using an equal volume of 0.25M glycine in PBS and pelleted. Cells were then resuspended in PBS containing 0.2% Triton-X100, 2% bovine serum albumin and 50 µg/ml RNAse A [Sigma], and incubated for 48 h. Tubulin was stained using mouse anti-tubulin-FITC [Sigma] and analysed by flow cytometry.

### 2.3. T cell transfer colitis

Female Balb/C and C.B-17 SCID animals were obtained from Harlan [Boxmeer, The Netherlands] and used between the ages of 8 and 20 weeks. Within each experiment, animals were within a 1 week age range. Animals were housed under SPF conditions in IVF cages in groups of 5. They were maintained on a 12-h light/dark cycle and provided with water and chow *ad libitum*. The T cell transfer model has been described previously.^8^ Briefly, wild type CD4+CD45RB^high^ cells are isolated from the spleen using magnetic depletion and flow cytometric cell sorting; 2.5 x 10E5 cells were transferred into C.B-17 SCID animals by intraperitoneal [i.p.] injection. Control animals received no T cell transfer. Three weeks after transfer, endoscopy was performed to ensure the onset of disease. Animals were randomised using an online randomiser [www.randomizer.org]. Treatment was allocated per cage because of possible faecal-oral transfer of albendazole. Treatment consisted of 1–100 µg/animal [titration experiment] or 25 µg/animal [combination treatment experiment] anti-murine TNF or IgG [both kindly provided by Dr D. Shealy, Janssen Research & Development]. For combination treatment, either 4 mg/animal albendazole [Sigma] in 0.5% carboxymethylcellulose or vehicle control was administred. This dose has been described previously for i.p. administration.^9^^,^^10^ All treatments were administered twice weekly by i.p. injection. Weight was monitored three times per week. In the combination treatment experiment, two animals were lost to follow-up [1 placebo, 1 albendazole monotherapy] due to complications upon endoscopy and subsequent premature sacrifice, and were excluded from the analysis. Upon sacrifice, disease activity index [DAI] was determined by the level of diarrhoea [score 0–3], colonic inflammation/oedema [0–3], and weight loss [0–4] as previously described. All scoring was performed by a blinded observer. All animal studies were approved by the local animal ethical committee, and performed according to national guidelines.

### 2.4. Histology

The primary outcome of the animal model is the histological scoring of colitis. Upon sacrifice, the colon was opened, washed, and cut longitudinally in half, and one part was processed for histology. Tissue was rolled into swiss rolls and routinely embedded in paraffin. Six-µm sections were cut and stained by haematoxylin and eosin [HE] staining. Sections were then blinded and randomised and scored for pathology, including the following parameters: immune infiltrate [0–3], goblet cell loss [0–3], crypt loss [0–3], crypt hyperplasia [0–3], and the presence of crypt abscesses [0 = no, 3 = yes]; total histological scoring was the combined score [0–15].

### 2.5. Quantitative polymerase chain reaction

Upon sacrifice, colon sections of approximately 10 mm were snapfrozen. RNA was isolated using the Isolate II RNA kit [GC Biotech, Alphen aan den Rijn, The Netherlands] and cDNA was generated using RevertAid reverse transcriptase [Thermo Scientific, Waltham, MA] and random primers [Promega, Leiden, The Netherlands]. Quantitative real-time polymerase chain reactions [RT-PCR] were carried out using Sensifast SybrGreen [GC Biotech]. For relative expression, all data were normalised against expression of the household gene cyclophilin, according to the 2^ddCt method.

### 2.6. Immunofluorescence

The MLR was performed in wells containing poly-L-lysine [Sigma] coated coverslips. Cultures were fixed using 4% PFA, permeabilised using 0.1% Triton X, and stained using αPhospho-AMPK [#2535S, Cell signaling, Beverly, MA], anti-CD14-FITC [BD Biosciences], and Goat-αRabbit-AF647 [Invitrogen, Carlsbad, CA]. Samples were embedded in SlowFade Gold containing DAPI [Invitrogen] and imaged using a Leica DM6000B microscope. Random images were taken by a blinded researcher, and analysed using ImageJ software [http://imagej.nih.gov/ij/],;pAMPK staining was normalised against DAPI.

### 2.7. *In situ* hybridization

Sections were cut freshly and dried overnight at 37°C. *In situ* hybridisation was performed using the RNAscope Reagent 2.5HD Red system [Advanced Cell Diagnostics, Milan, Italy], using probes Mm-Ifng, Mm-Il17a, and Mm-IL10. After development of the FastRed signalling, sections were washed once in tap water, and twice in PBS. Sections were then stained using anti-CD3 [Dako, Copenhagen, Denmark] and Goat-αRabbit-AF488 [Invitrogen] and mounted in SlowFade Gold containing DAPI [Invitrogen] and imaged using a Leica DM6000B microscope. Images were analysed using ImageJ software [http://imagej.nih.gov/ij/]. CD3 positive staining was calculated relative to DAPI staining. T cell skewing was calculated as the number of CD3+ cells containing at least two positive spots, as per the manufacturers suggestion.

### 2.8. Statistics

Data were analysed using GraphPad Prism [Graphpad Software Inc., La Jolla, CA]. Statistical analysis included one-way analysis of variance [ANOVA] followed by *post hoc* testing [Newman Keuls] and Kruskal-Wallis followed by Dunns *post hoc* analysis for quantitative PCR data. All bars represent means, with error bars indicating standard error of the mean [SEM]. Data were considered significant when *p* < 0.05.

### 2.9. Ethical statement

All animal studies were approved by the institutional animal ethical committee and performed according to national guidelines.

## 3. Results

### 3.1. Benzimidazole anti-helminthics enhance the induction of regulatory macrophages by anti-TNF

**Figure 1.**
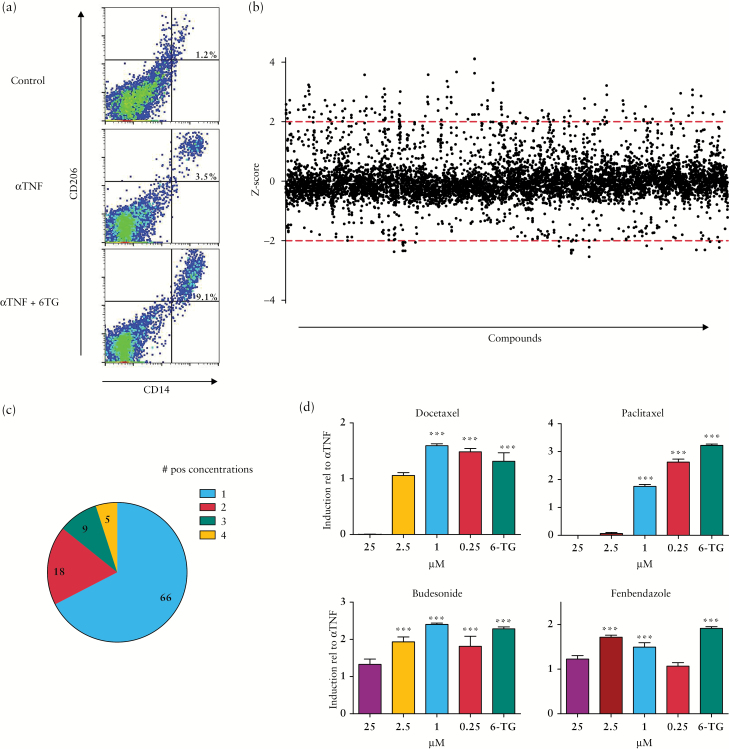
Library screen reveals potential of anti-helminthics to enhance the induction of regulatory macrophages by anti-TNF. [A] Induction of CD14+CD206+ macrophages in MLR in 96 well plates [Control IgG, IFX 10 µg/ml, and IFX + 6TG 25 µM]. [B] Z-scores of all compounds tested in screen at each of four concentrations. Each dot represents average of duplicate samples. Dotted lines represent Z-scores of 2 [top line] and -2 [bottom line], which were used as cut-off values. [C] Number of compounds reaching Z-scores > 2 divided by number of positive concentrations. [D] Validation of compounds in a secondary laboratory. Induction of CD14+CD206+ macrophages was calculated as relative to induction by IFX alone in the same culture. Bars represent mean, error bars represent standard error of the mean., *n* = 5/sample. ***p < 0.001. TNF, tumour necrosis factor; MLR, mixed lymphocyte reactions; IFX, infliximab.

Using a MLR with human peripheral blood mononuclear cells as previously described, we screened the Pharmakon 1600 library for drugs with the capacity to enhance the anti-TNF mediated induction of CD14+CD206+ regulatory macrophages. Each compound was tested at four different concentrations [25, 5, 1, and 0.2 µM]. As a positive control, 6-thioguanin, a metabolite of the thiopurine azathioprine, was included on each plate [Figure 1a; and Figure S1a, available as Supplementary data at *ECCO-JCC* online]. Of all compounds tested, 98 compounds significantly enhanced anti-TNF mediated induction of regulatory macrophages at at least one concentration [Figure 1b/c and Table S1, available as Supplementary data at *ECCO-JCC* online]. Notably, 6-thioguanin is one of the compounds present in the library and was one of the positive hits in the screen, validating the approach. Several of the positive compounds clustered in a few functional classes, such as steroids and cytostatic drugs [Supplementary Figure S1b, available as Supplementary data at *ECCO-JCC* online]. Remarkably, six out of nine benzimidazoles present in the library tested positive in the screen, whereas the closely related [nitro]imidazoles tested negative.

**Figure 2.**
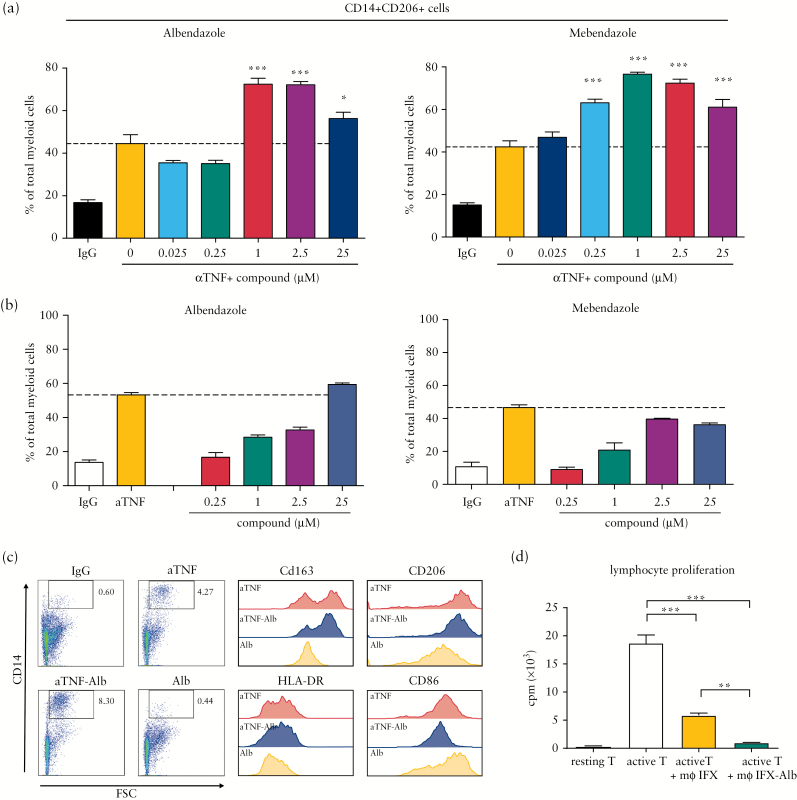
Validation of benzimidazoles as co-medication to anti-TNF *in vitro*. [A,B] Extended dose titration of albendazole and mebendazole on the induction of CD14+CD206+ macrophages in the presence [A] or absence [B] of IFX [10 µg/ml]. Dotted line indicates induction by IFX alone. [C] Phenotypic analysis of macrophages induced in the presence of IFX and/or albendazole [1 µM]. Dot plots represent total cultures, histograms have been gated for CD14+ cells. Data shown are obtained from a single experiment and representative of three experiments using two individual donors/experiment. [D] Assessment of immunosuppressive capacity of combination treatment-induced macrophages. Macrophages were induced in the MLR in the presence of IFX and/or albendazole [25 µM], isolated based on CD14 expression. and plated in equal numbers. Activated third-party lymphocytes were added and proliferation measured by 3H thymidine incorporation. Data representative of one of three experiments, *n* = 5/condition. Bars represent mean, error bars represent s.e.m. *p < 0.05; **p < 0.01; ***p < 0.001. TNF, tumour necrosis factor; IFX, infliximab; MLR, mixed lymphocyte reactions; s.e.m., standard error of the mean.

To confirm the results obtained in the large-scale screen, 15 compounds from various drug classes were re-acquired from a second vendor and tested in a secondary assay. Compounds from various drug classes were selected based on clinical applicability. Of these 15 compounds, 13 indeed proved to enhance the macrophage-inducing capacity of anti-TNF, including the cytostatic agents docetaxel and paclitaxel, the steroid budesonide, and the anti-helminthics fenbendazole, albendazole, and mebendazole [Figures 1d and 2a; Table S2, available as Supplementary data at *ECCO-JCC* online]. In view of the favourable safety profile and high tolerability of benzimidazoles and the novelty of their immunomodulatory potential, we further investigated the effects of benzimidazoles.

### 3.2. Macrophages induced in the presence of anti-TNF and benzimidazoles show regulatory phenotype and function

Dose titration studies for albendazole and mebendazole revealed that, for both compounds, doses in the range 1–25 µM enhanced anti-TNF mediated macrophage induction [Figure 2a]. Due to the complexity of the analysis, the original screen only tested compounds in the presence of anti-TNF, but not as monotherapy. Further analysis in the absence of anti-TNF showed that although each compound induced CD14+CD206+ cells in the high-dose range [Figure 2b], this induction did not surpass the induction seen by anti-TNF alone and was lower than the induction seen after combination treatment. Phenotypic analysis showed that macrophages induced by anti-TNF plus albendazole combination treatment were very similar to macrophages induced with anti-TNF alone. Both were characterised by expression of the M2-associated markers CD206 and CD163, intermediate levels of HLA-DR, and low levels of CD86 [Figure 2c]. In contrast, macrophages induced with albendazole alone expressed lower levels of CD163 and CD206 and higher levels of CD86, consistent with a less regulatory phenotype. To confirm that the macrophages induced by albendazole plus anti-TNF have regulatory function, CD14+ cells were isolated from MLR treated with this combination and were cultured with third-party activated lymphocytes. We have previously shown that anti-TNF induced regulatory macrophages suppress T cell proliferation in this set-up.^5^ Macrophages induced with anti-TNF/albendazole combination treatment inhibited T cell proliferation to a greater extent than equal numbers of macrophages induced with anti-TNF alone [Figure 2d]. Thus, albendazole not only increases the number of M2 macrophages induced by anti-TNF, but also potentiates their immunosuppressive properties. To test the skewing potential of albendazole in the classic M1/M2 differentiation protocols, monocytes were cultured in the presence of IFNγ, IL-4, and/or anti-TNF and albendazole. Although anti-TNF plus albendazole treatment of IFNγ-induced macrophages resulted in a more M2-like morphology and minor modulation of surface markers, the effect was marginal compared with the effect of the cytokine stimulation [Supplementary Figure S2, available as Supplementary data at *ECCO-JCC* online].

### 3.3. Albendazole-dependent potentiation of macrophage induction is mediated through the cytoskeletal-AMPK axis

**Figure 3.**
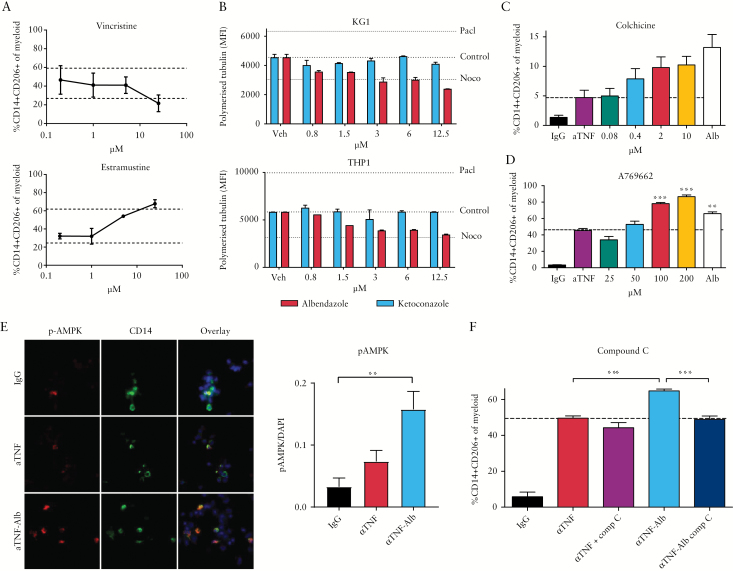
Albendazole enhances anti-TNF mediated macrophage induction through tubulin binding and AMPK signaling. [A] Re-analysis of the original screen data indicates a trend for other tubulin-binding compounds in induction of CD14+CD206+ macrophages in the presence of IFX. Graphs show mean of duplicate samples in the original screen, lower dotted line indicates induction by IFX alone [lower line] or IFX plus 6-TG [upper line] on the same plate. [B] KG1 and THP1 cell lines were treated with albendazole and ketoconazole in concentrations indicated, and polymerised tubulin was measured by flow cytometry. Dotted lines indicate maximum polymerisation using paclitaxel [25 nM], control [DMSO only], and minimum polymerisation after inhibition using nocodazole [2 µM]. [C,D] Induction of CD14+CD206+ macrophages in the presence of IFX [10 µg/ml] and colchicine [C] and A769662 [D] in concentrations indicated. Albendazole [1µM] was included for reference purposes, dotted line indicates induction by IFX alone. Graph depicts an individual experiment [B, *n* = 5/sample; C, *n* = 6/sample] representative of three independent experiments. [E] MLR were performed on coverslips treated with IFX and/or albendazole [1µM] and stained for phospho-AMPK [red], CD14 [green], and DAPI [blue]. Micrographs depict representative images, graph represents quantification of > 5 images/condition. [F] Effect of the AMPK inhibitor compound C on CD14+CD206+ macrophage induction. Compound C [0.5 µM] was added to cultures treated with IFX and/or albendazole [1µM]. Plots depict individual representatives, graphs depict one of three independent experiments [n = 6/condition]. Bars represent mean, error bars represent s.e.m. **p < 0.01, ***p < 0.001. TNF, tumour necrosis factor; IFX, infliximab; MLR, mixed lymphocyte reactions; s.e.m., standard error of the mean.

Benzimidazoles, including albendazole, exert their anti-helminthic effects through inhibition of nematode tubulin and subsequent inhibition of energy metabolism. Although affinity for helminthic tubulin is reported to be significantly higher than that for mammalian tubulin, polymerisation of the latter is also inhibited by these compounds.^11,12^ Closer analysis of the data obtained in the screen revealed that two other tubulin-modifying drugs [vincristine and estramustine] also tended to increase anti-TNF mediated induction of regulatory macrophages, suggesting tubulin modulation as a potential mechanism [Figure 3a]. Indeed, albendazole [but not ketoconazole] inhibited tubulin polymerisation in two myeloid cell lines [Figure 3b]. Benzimidazoles have been shown to interact with tubulin at a colchicine-binding site, and colchicine also showed a potentiating effect on anti-TNF mediated macrophage induction [Figure 3c]. It has previously been shown that colchicine can skew macrophages to an M2 phenotype, and that this is dependent on AMPK signalling.^13^ Indeed, we found that the specific AMPK activator A-769662 fully recapitulated the effect of albendazole on anti-TNF mediated macrophage induction [Figure 3d]. Immunofluorescence indicated that AMPK phosphorylation occurred mainly in CD14+ cells within the culture, and was strongly upregulated in MLR after anti-TNF/albendazole combination therapy but only to a limited extent in cultures treated with anti-TNF alone [Figure 3e]. In line with a central role for AMPK signalling in the stimulatory effect of albendazole on regulatory macrophage development, inhibition of AMPK using compound C completely abrogated the additive effect of albendazole on macrophage induction, and reduced the percentage of regulatory macrophages to that generated with anti-TNF alone [Figure 3f].

### 3.4. Albendazole enhances the therapeutic effect of anti-TNF in T cell transfer model of colitis

Anti-TNF and albendazole combination therapy was further studied *in vivo* using the T cell transfer model of colitis. The commonly used dose of anti-TNF in murine models ranges around 100–300 µg/mouse twice weekly. This dose results in near complete disease remission after 4 weeks of treatment,^14^ negating the possibility of testing drugs that potentiate the effect of anti-TNF. We therefore first titrated the anti-mouse TNF to identify a dose that could be considered suboptimal. As expected, administration of 100 µg/animal twice weekly resulted in near complete healing of the intestine in all animals after 4 weeks of treatment [Supplementary Figure S3, available as Supplementary data at *ECCO-JCC* online]. In contrast, 25 µg/animal twice weekly resulted in an intermediate response, both in body weight and in histological disease, and lower doses had no effect. We thus proceeded to test the efficacy of the combination therapy using an anti-TNF dose of 25 µg/animal twice weekly.

**Figure 4.**
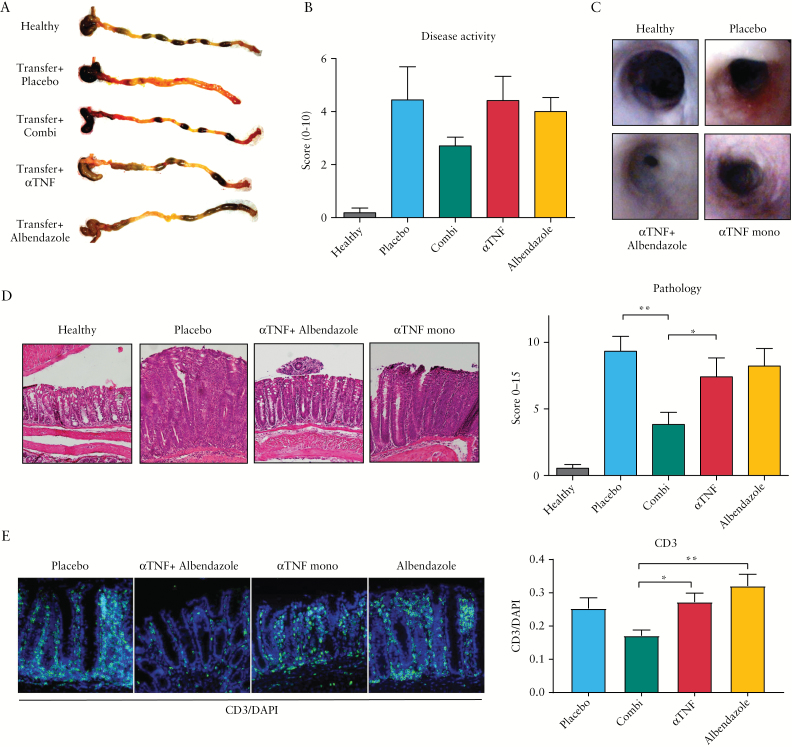
Albendazole improves the response to anti-TNF in murine transfer colitis. Colitis was induced by transfer of CD45RB^high^ CD4+ T cells into CB17.SCID animals. Starting 3 weeks after transfer, animals were treated using anti-TNF or isotype control [both 25 µg/animal twice weekly] and albendazole [4 mg/animal twice weekly] or vehicle control. [A] Examples of colon of each group are depicted, healthy *n* = 11, placebo *n* = 10, anti-TNF *n* = 12, combination *n* = 12, albendazole *n* = 9. [B] Disease activity at the end of treatment. [C] Endoscopic images taken at Day 35 [2 weeks of treatment]. Images representative for each group. [D] Histopathological analysis of colonic tissue after 2 weeks of treatment. Photographs depict representative images, magnification 100x, graph depicts summarised data of all animals. Bars represent mean, error bars represent s.e.m. [E] Analysis of CD3+ cells in murine colitis model. CD3+ cells were visualised by immunofluorescence [magnification 100x] and presence of CD3+ staining relative to total number of cells [represented by DAPI] was calculated.*p < 0.05; **p < 0.01. TNF, tumour necrosis factor; s.e.m., standard error of the mean.

As expected with this suboptimal dosing scheme, endoscopic and histological analysis clearly showed remaining inflammation in the animals on anti-TNF monotherapy [Figure 4a-d]. In contrast, animals receiving anti-TNF plus 4 mg/animal albendazole combination therapy showed significant improvement in the severity of their colitis, both compared with placebo and compared with anti-TNF alone. Both treatments reduced weight loss [Supplementary Figure S4a, available as Supplementary data at *ECCO-JCC* online], in line with previous data indicating that prevention of body weight loss is mediated mainly through neutralisation of systemic TNF, whereas intestinal healing requires additional effector mechanisms.^14^ Albendazole monotherapy did not improve colitis significantly. Supporting the hypothesis of a potentiating effect of albendazole on anti-TNF therapy rather than an independent immunosuppressive effect, levels of albendazole metabolites correlated significantly with disease activity in combination-treated animals [R = -0.62, *p* = 0.03], but not in the albendazole monotherapy group [R = -0.01, *p* = 0.98, Figure S4b].

Analysis of the T cell compartment confirmed a significantly decreased presence of CD3+ cells after anti-TNF plus albendazole combination treatment [Figure 4e]. Using combined immunofluorescence and *in situ* hybridisation, T cell polarisation was investigated. Within the CD3+ present, a trend to decreased IFNγ expression was observed both with anti-TNF alone and with combination treatment, whereas IL-17 was only detected in a minority of CD3+ cells [Supplementary Figure S3, available as Supplementary data at *ECCO-JCC* online]. These data suggest that the effects are mediated more though modulation of T cell numbers than through altered polarisation.

**Figure 5.**
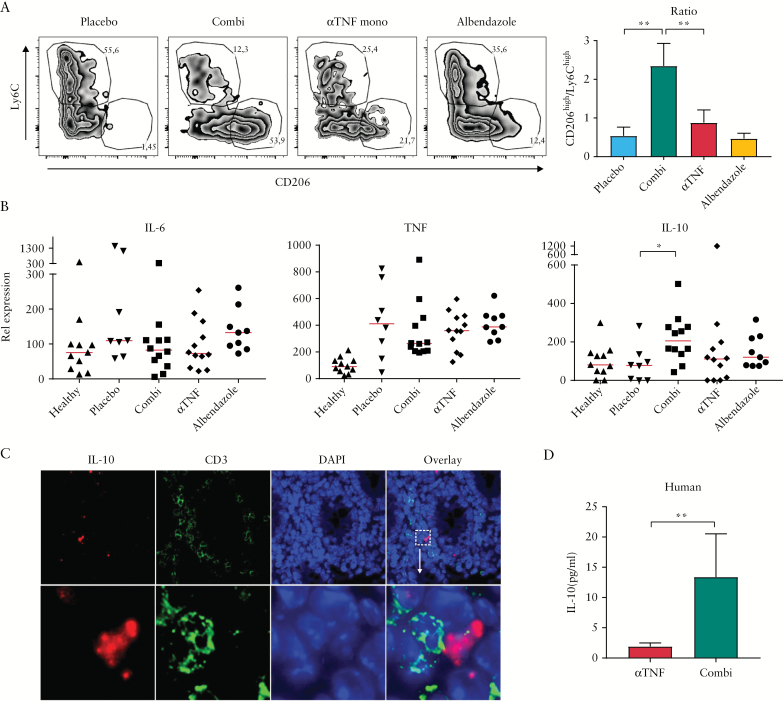
Albendazole increases the M2/M1 ratio and decreases CD3+ cells *in vivo*. [A] Colonic tissue was analysed by flow cytometry. Macrophages were gated as DAPI-CD45+Ly6G-CD11b+CD64+ cells and subdivided into M1-like [Ly6C^high^CD206^low^] and M2-like [Ly6C^low^CD206^high^] cells. Graph depicts summary of all animals, plots depict representative examples. Bar graph shows ratio between M2-like versus M1-like macrophages. [B] Cytokine levels in individual animals as determined by quantitative PCR. Expression is normalised to cyclophilin and represented relative to the average of the control group [set to 100%]. Lines indicate median. [C] Expression of IL-10 transcript was determined by fluorescent *in situ* hybridisation [red], with CD3 [green] as counterstain. Dotted square indicates enlargement shown in lower panel. [D] IL-10 secretion by human macrophages after incubation with anti-TNF or combination therapy. Bars represent mean, error bars represent s.e.m. *p < 0.05; **p < 0.01. PCR, polymerase chain reaction; s.e.m., standard error of the mean.

To investigate whether the therapeutic effect was associated with enhanced induction of regulatory macrophages *in vivo*, colonic tissue was analysed for macrophage subtypes. Anti-TNF plus albendazole combination-treated animals showed decreased presence of total macrophages [identified as CD45+CD11b+Ly6G-CD64+ cells, Figure S4c], consistent with data obtained in IBD patients upon response to anti-TNF.^4^ The macrophages present were further analysed for expression of Ly6C and CD206, which have been described to differentiate between immunosuppressive and inflammatory subsets.^15^ In placebo-treated animals, over half of macrophages were Ly6C^high^CD206^low^, consistent with an inflammatory phenotype [Figure 5a]. In contrast, upon combination treatment, the majority of macrophages displayed the more regulatory Ly6C^low^CD206^+^ phenotype. Again, this is consistent with data obtained previously in human IBD patients. Interestingly, the ratio of anti- versus pro-inflammatory macrophages correlated strongly with both disease activity [R = -0.54, *p* < 0.001] and histological scoring [R = -0.55, *p* < 0.001, Figure S3d].

Although missing significance, IL-6 appeared slightly increased in the albendazole-monotreated group, which was not observed in the combination-treated animals [Figure 5b]. In contrast, transcription of IL-10 was significantly increased in combination-treated animals, suggesting an active induction of regulatory mediators. The source of the IL-10 was further assessed using *in situ* hybridisation. Cells expressing high levels of IL-10 were mainly found in the mesenchymal compartment in close proximity to CD3-expressing cells, but did not express CD3 themselves [Figure 5c]. The induction of IL-10 by combination treatment was further corroborated in human macrophages, which showed increased secretion of IL-10 upon stimulation by anti-TNF and albendazole compared with anti-TNF alone [Figure 5d].

## 4. Discussion

Anti-TNF monoclonal antibodies induce regulatory macrophages *in vitro* and *in vivo* and these may play an important role in mucosal healing in patients with IBD. Thiopurines promote the induction of regulatory macrophages by anti-TNF, thus providing one potential mechanism of action of anti-TNF/thiopurine combination therapy. Using our previously described macrophage induction model as a screening tool, we now identify benzimidazoles as drugs that promote regulatory macrophage phenotype in an AMPK-dependent manner. The commonly used benzimidazole albendazole not only enhanced anti-TNF induced regulatory macrophage development, but also potentiated the therapeutic effect of an anti-TNF in the T cell transfer model of colitis.

Classically, macrophages have been described as pro-inflammatory ‘M1’ and regulatory ‘M2’ type cells. These cell types were usually generated in the presence of excessive amounts of exogenous cytokines, and most likely represent extremes of the total spectrum occurring *in vivo*, with most macrophages presenting as intermediate and plastic phenotypes.^16^ In addition, macrophage responses may depend on type, dosing, and timing of stimulation. Nonetheless, the extremes of the M1/M2 model illustrate the vast scope of functions macrophages can exert, ranging from strongly pro-inflammatory cells producing reactive oxygen species and recruiting other immune cells, to immunosuppressive cells involved in resolution of immune responses and wound healing. We have previously shown that anti-TNF induces highly immunosuppressive CD14+ CD163+ CD206+ macrophages which promote epithelial wound healing.^4^^,^^5^ In a cohort of IBD patients, the induction of such macrophages correlated with endoscopic response to infliximab. Intriguingly, we found that the development of anti-TNF induced regulatory macrophages was stimulated by thiopurines, which provides a potential mechanism of action of the additive effect of azathioprine to infliximab in IBD therapy.

Unfortunately, a relatively large proportion of IBD patients are intolerant to thiopurines and in this group, anti-TNF/thiopurine combination therapy is not possible.^6^ In an attempt to identify an alternative for this combination therapy, we used the induction of regulatory macrophages as a screening tool and analysed the Pharmakon 1600 library for compounds that promoted anti-TNF mediated development of regulatory macrophages. All compounds in this library have reached clinical evaluation and demonstrated biological activity against known targets, making rapid clinical evaluation possible for drug repurposing. The screen identified benzimidazoles as drugs that potently enhance anti-TNF induced regulatory macrophage development. Benzimidazoles are anti-helminthic drugs, mainly used for the eradication of pinworms and the treatment of cystic echinococcosis. In particular in tropical helminthic infections, usage up for to 3 months has been reported and was associated with relatively mild and self-limiting adverse events.^17,18^ Their mechanism of action has been attributed to a direct toxic effect on nematodes,^19^ but our data show that these compounds also induce M2-like macrophages, even in the absence of anti-TNF. As M2 macrophages play a key role in the defence against parasites in the gut,^20^ the stimulation of the M2 macrophage phenotype may be an additional mechanism of action of benzimidazoles as anti-helminthic drugs.

The initial rationale behind the concomitant use of anti-TNF and immune modulators such as thiopurine was to prevent the generation of anti-drug antibodies which are associated with loss of response.^21^ Indeed higher trough levels of infliximab were seen in patients receiving anti-TNF plus thiopurine combination therapy,^22^ and subsequent studies showed a decreased level of anti-drug antibodies.^23^ However, beneficial effects of this combination therapy were seen even after stratification for the presence of anti-drug antibodies, suggesting additional modes of action. One potential additional mechanism is indicateded by our previous data, showing enhanced induction of regulatory macrophages upon anti-TNF plus thiopurine combination therapy. The role of regulatory macrophages is further emphasised in the current study, as anti-TNF plus albendazole combination therapy also results both in increased numbers of regulatory macrophages and in increased clinical response to anti-TNF. Importantly, in the animal model used here, animals are devoid of B cells and therefore do not produce anti-drug antibodies, excluding this as a mechanism of action in this particular model.

Due to the large number of compounds included in the screen, the current data also provide clues regarding the biological mechanism of regulatory macrophage induction. Benzimidazoles enhanced this induction in the presence of anti-TNF, but the related families of imidazoles and nitroimidazoles did not. Benzimidazoles exert their anti-helminthic effect through modulation of helminthic tubulin, but also interfere with mammalian tubulin polymerisation.^11^ Supporting a role for tubulin binding as an effector mechanism, other tubulin-modulating drugs also proved effective in potentiating the development of anti-TNF induced macrophages. In addition, albendazole resulted in decreased tubulin polymerisation in myeloid cells whereas ketoconazole, which does not potentiate macrophage induction, had no effect. Interestingly, others have demonstrated the induction of a more pro-inflammatory phenotype of macrophage using mebendazole.^24^^,^^25^ In those experiments, mebendazole increased secretion of IL1b and TNF through activation of the ERK pathway. In our hands, ERK inhibition did not abrogate the macrophage-inducing effects of albendazole [data not shown]. These differences may be due to either the specific compound used [mebendazole versus albendazole], the use of a cell line versus primary cells, or the specific context of anti-TNF treatment in our experiments.

Downstream of these events, benzimidazoles induced AMPK activation, whereas inhibition of AMPK activity completely abrogated the effect of albendazole. AMPK is a highly conserved enzyme composed of three subunits, of which the alpha subunit contains the catalytic activity. AMPK mainly functions as an energy sensor, and is activated by phosphorylation upon decreasing levels of intracellular ATP. Through a multitude of intermediates,^26^ activated AMPK inhibits energy-consuming processes such as fatty acid and cholesterol synthesis, and activates catabolic mechanisms such as glucose and fatty acid uptake as well as fatty acid oxidation.^27^ This same shift in metabolic activity occurs during differentiation of M2-type macrophages. Conversely, inhibition of oxidative phosphorylation increases secretion of the pro-inflammatory cytokines IL6 and IL-12.^28^^,^^29^ In line with this, a role for AMPK in M2 polarisation has been described before. Macrophage-specific AMPK-/- animals fail to generate M2-type macrophages after muscle injury, and subsequently display delayed tissue regeneration.^30^ In microglia, a brain tissue-specific type of macrophage, AMPK signalling is critically important for expression of IL-10 after stimulation. Interestingly, deletion of AMPK signalling also inhibited expression of CD206 in these cells. We observed an increased expression of IL-10 and CD206 upon combination therapy, both *in vivo* and *in vitro*. The immunosuppressive effects of regulatory macrophages appeared mediated through general suppression of T cell responses, as decreased numbers of CD3+ cells were present in the colons after combination therapy, whereas T cell polarisation was not affected compared with anti-TNF therapy alone. These data strongly suggest the effect of anti-TNF+albendazole combination therapy is mediated through altered macrophage skewing, although we cannot exclude additional effects on other cell types *in vivo*.

In summary, these data highlight the importance of regulatory macrophages as a therapeutic target in IBD, and show for the first time that benzimidazoles promote development of anti-TNF induced regulatory macrophages. This is an important step towards the repurposing of this well-established, safe, and widely available drug class for the treatment of IBD.

## Funding

This study was supported by the European Crohn’s and Colitis Organisation [ECCO grant 2015 to MW] and the Dutch Society for Gastroenterology [NVGE Gastrostart 2013 to GB].

## Conflict of Interest

MEW reports non-financial support from Janssen, during the conduct of the study; and personal fees from Takeda, outside the submitted work. AC is currently an employee of Adaptimmune. PJK reports personal fees from Merck Sharp and Dohme, outside the submitted work. FMB reports grants from AbbVie, grants from Janssen Prevention Center of Janssen Vaccines & Prevention BV, Leiden, The Netherlands, part of the Janssen Pharmaceutical Companies of Johnson & Johnson, outside the submitted work. JFB reports personal fees from Abbvie, personal fees from Merck Sharp en Dohme, and personal fees from Takeda,outside the submitted work. GD’H reports grants from MSD, Abbot, GSK, personal fees from MSD, Abbot, Shire, personal fees from Ablynx, grants and personal fees from Abbvie, personal fees from Amakem, personal fees from AM Pharma, personal fees from BMS, personal fees from Boehringer Ingelheim, personal fees from Celgene, personal fees from Covidien, grants and personal fees from Ferring, grants from DrFALK Pharma, personal fees from Celltrion, grants and personal fees from Centocor/Jansen Biologics, personal fees from Engene, personal fees from Galapagos, personal fees from GSK, personal fees from Hospira, personal fees from Medimetrics, grants and personal fees from Millenium/Takeda, personal fees from Mitsubishi Pharma, grants and personal fees from MSD, grants and personal fees from Mundipharma, personal fees from Pfizer, grants from Photopill, grants and personal fees from Prometheus laboratories, personal fees from Receptos, personal fees from Robarts Clinical Trials, personal fees from Salix, personal fees from Sandox, grants and personal fees from Setpoint, personal fees from Shire, personal fees from TEVA, and personal fees from Tillots, outside the submitted work. GRB is currently an employee of GSK and reports grants from AbbVie, personal fees from AbbVie, personal fees from MSD, grants from Crucell BV, and from Takeda, outside the submitted work. All other authors report nothing to disclose.

## Author Contributions

MW, AD, AC, and DE designed, performed, and analysed the screen experiments. MW, AD, ZG, and PK performed *in vitro* validation experiments. MW, FB, JB, and PK performed *in vivo* experiments. TH performed and analysed HPLC experiments. AS, GD, DE, LW, and GB supervised the study and provided clinical input. MW, LW, and GB wrote the manuscript, with input from all authors.

## Supplementary Material

Supplementary_information
