# Supplementary material for: Benzimidazoles Promote Anti-TNF Mediated Induction of Regulatory Macrophages and Enhance Therapeutic Efficacy in a Murine Model
Source: J Crohns Colitis. 2017 Aug 16;11(12):1480–90. doi: 10.1093/ecco-jcc/jjx104 (PMC5881671; doi:10.1093/ecco-jcc/jjx104)
Supplement: Supplementary_information [file jjx104_suppl_supplementary_information.pdf]

Supplementary methods

Compound library screen

PBMC were isolated from healthy donor buffy coats as described above. Cells from 4 individual donors were mixed in equal numbers and plated into 96-well U bottomed plates (total 2x10E5 cells/well). Cells were cultured in RPMI containing 10% FCS, L-Glutamine, penicillin for 2 days at 37°C and 5% CO_2. As controls, anti-TNF (Infliximab), control IgG (both 10 µg/ml) and/or 6-thioguanin (6-TG, Sigma) were added to multiple wells on each plate. The Pharmakon 1600 drug library was added at 4 final concentrations (0.2 µM, 1 µM, 5 µM, 25 µM). Each combination of anti-TNF plus compound was run on duplicate plates. Liquid handling was performed using the Flexdrop Reagent dispenser and a Janus automated workstation equipped with 96-well MDT (both Perkin Elmer, Waltham, MA). After 4 days, cells were washed, detached using PBS containing 5mM EDTA, stained using anti-CD14-FITC (clone MϕP90) and αCD206-APC (clone 19.2) and fixed in 1% PFA. Samples were measured using a FACS LSRII equipped with High Throughput Sampler and analysis was performed using FlowJo Software (Treestar Inc., Ashland, OR). Samples resulting in less than 200 analyzable cells were excluded (256 samples, 2%). In the majority of cases, exclusion was most likely caused by cytotoxicity, and mainly included samples treated with 25 µM of compounds. For overall screen performance, Z-factors of individual plates were calculated based on anti-TNF and anti-TNF plus 6-TG controls. Average Z-factor was 0.33 (95% CI 0.29-0.38). Z scores were calculated for averaged duplicates as has been described earlier[8]. Using a Z score of 2, anti-TNF and anti-TNF plus 6-TG combination could be discriminated with a sensitivity of 93% and a specificity of 100%. Therefore, a Z score of 2 was used as cut-off for determination of positive hits from the screen.

Analysis of colonic macrophages

For analysis of colonic tissue, colons were removed and longitudinally cut in half. One part was used for flow cytometry, the other for histological assessment. Colon sections were cut into 0.5 cm pieces and washed three times in PBS. Subsequently tissue sections were incubated in HBSS supplemented with 2% FCS and 5mM EDTA for 20 minutes while shaking. Cells and remaining tissue were pelleted, minced very fine and incubated in HBSS supplemented with 2% FCS, 5mg/ml Liberase TL and 10 μg/ml DNAse (both Sigma Aldrich) for 40 minutes while stirring. Suspensions were then passed through a 100 μm cell strainer, pelleted and stained for flow cytometry in PBS containing 1% BSA. Antibodies used were obtained from Biolegend: αCD45-APC-Cy7 (clone 30-F11), αCD11b-PerCP (clone M1/70), αCD64-PE (clone X54-5/7.1), αCD206-AF488 (clone MR5D3), from BD Bioscience: αLy6G-AF700 (clone 1A8), and from e-Bioscience: αLy6C-APC (clone HK1.4). Colonic macrophages were defined as CD45+CD11b+Ly6G-CD64+. Only samples with >100 macrophages were included, which resulted in exclusion of 3 samples (1 placebo, 1 anti-TNF monotherapy, 1 combination therapy).

Measurement of serum albendazole concentrations

Plasma samples were collected in heparinized tubes and stored at -80 °C. The plasma samples were deproteinized with acetonitrile (3 volumes) on ice (5 minutes) followed by centrifugation in a tabletop Eppendorf centrifuge (10 minutes 14000 x g at 4 °C).

For the separation of albendazole and metabolites, a BDS Hypersil C18 column (150 x 4.8 mm, 3 μm particles, Thermo Scientific) at 25 °C was used. Flowrate was 1.0 ml/min, solvent A: 7 mM ammoniumformiate pH3.9, a linear gradient with solvent B: 100% acetonitrile and fluorescence

detection (excitation 280nm, emission 340nm). Standard curves for albendazole, albendazole-sulfon and albendazole-sulfoxide (Fluka) were used to determine the concentrations in plasma.

Supplementary figure legends

Supplementary figure 1. (A) Z-score of controls included in the screen. Each plate contained control samples (n= 4 IFX only and n= 4 IFX+6TG 25 µM). Z factors were calculated as averages of duplicate plates. Dotted line indicates cut-off value as used in the screen. (B) Heatmap representing Z-scores of all samples in each of four concentrations.

Supplementary figure 2. Modulation of M1/M2 polarization by anti-TNF and albendazole. Primary human monocytes were polarized in the presence of IFNγ (20 ng/ml) or IL-4 (10 ng/ml) and treated with anti-TNF (10 µg/ml), albendazole (25µM) or both for 48 hours. (A) DIC imaging of macrophages under various conditions (magnification 200x). (B) Flow cytometric analysis of M1/M2 markers. Bars represent mean, error bars represent s.e.m.

Supplementary figure 3. Dose titration of murine anti-TNF therapy in transfer colitis. Colitis was induced by transfer of $CD45RB^{high}$ CD4+ T cells into CB17.SCID animals. Starting three weeks after transfer, animals were treated using anti-TNF in dosages indicated or vehicle control. Dosing shown per animal 2/weekly. (A) Weight was monitored and shown as percentage relative to the first day of treatment. (B,C) Disease activity and histological scoring was determined at day 49 (4 weeks of treatment). Groups consisted of 11-12 animals (Healthy n=12, placebo n=12, 1 µg n=12, 5 µg n=11, 25 µg n=12, 100 µg n=11). Bars represent mean, error bars represent s.e.m. * p<0.05, **p<0.01, *** p<0.001, all compared to placebo.

Supplementary figure 4. Colitis was induced by transfer of $CD45RB^{high}$ CD4+ T cells into CB17.SCID animals. Starting three weeks after transfer, animals were treated using anti-TNF or isotype control (both 25 ug/animal twice weekly) and albendazole (4 mg/animal twice weekly) or vehicle control. (A) Weight was monitored as percentage relative to the first day of treatment. (B)

Serum levels of the active albendazole metabolite albendazole-sulfone (ABZSO2) were determined by HPLC and correlated to disease activity. Each dot represents an individual animal. (C) Colonic single cell suspensions were analyzed by flow cytometry. Total presence of immune cells was determined as CD45+ cells among all DAPI- cells, presence of macrophages was determined as CD11b+CD64+ cells within CD45+ population. Each dot represents an individual animal.(D) Ratio of M2/M1 macrophages was determined by flow cytometry and correlated to disease activity and histological disease score. Each dot represents an individual animal.

Supplementary figure 5. Analysis of T cell subsets in the murine colitis model. Colitis was induced by transfer of $CD45RB^{high}$ CD4+ T cells into CB17.SCID animals. Starting three weeks after transfer, animals were treated using anti-TNF or isotype control (both 25 ug/animal twice weekly) and albendazole (4 mg/animal twice weekly) or vehicle control. Expression of IFNγ (red,top) and IL-17 (red,bottom) were determined by fluorescent in situ hybridization and CD3 (green) was detected by immunofluorescence. Proportion of CD3+ cells expressing cytokine was determined by image analysis. Representative image shown, graph depicts summary of experiment (n>10 images/condition).

Fig S1

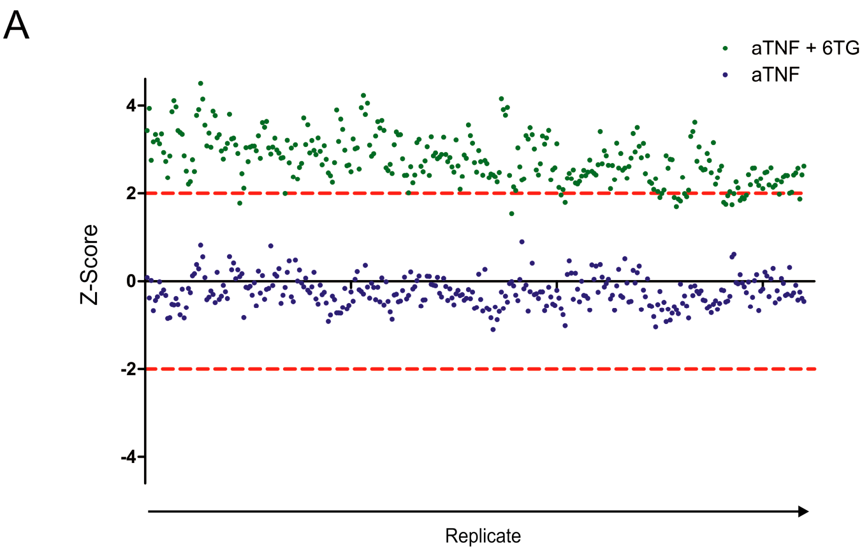
A
aTNF + 6TG
aTNF
Z-Score
4
2
0
-2
-4
Replicate

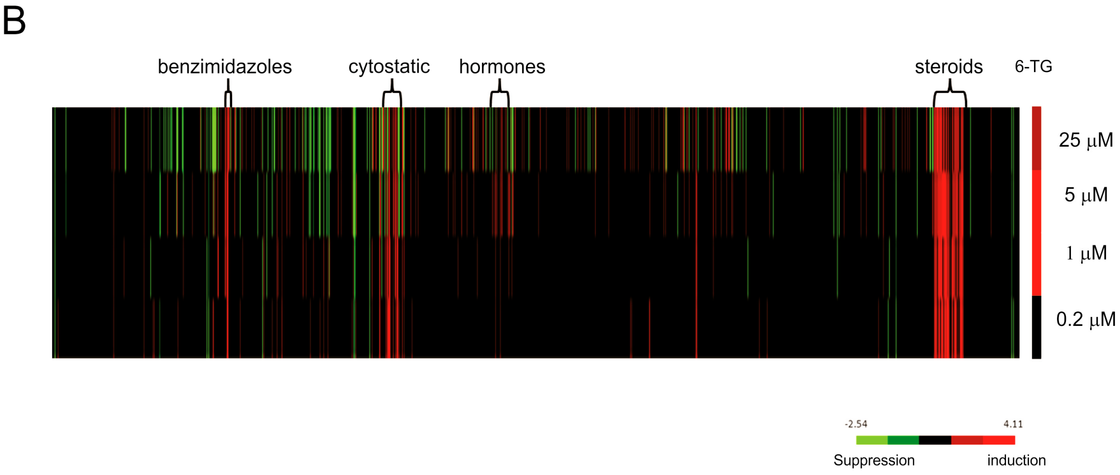
B
benzimidazoles
cytostatic
hormones
steroids
6-TG
25 μM
5 μM
1 μM
0.2 μM
-2.54
4.11
Suppression
induction

Fig S2

A

Control IFNγ IL-4

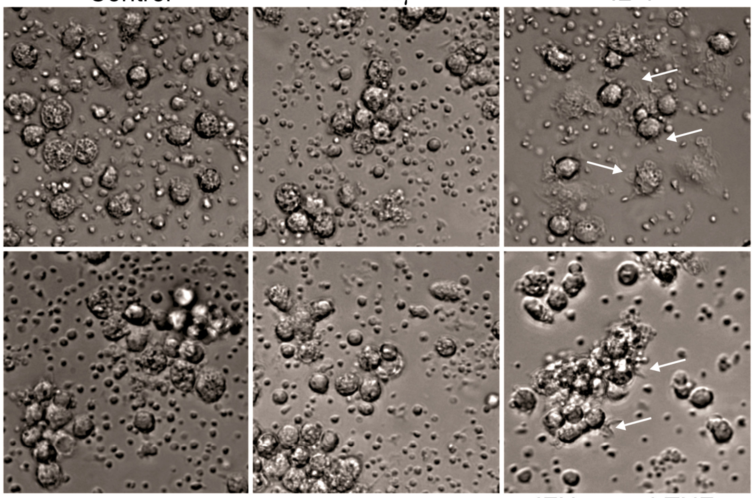

IFNγ + antiTNF IFNγ + albendazole IFNγ + anti-TNF + albendazole

B

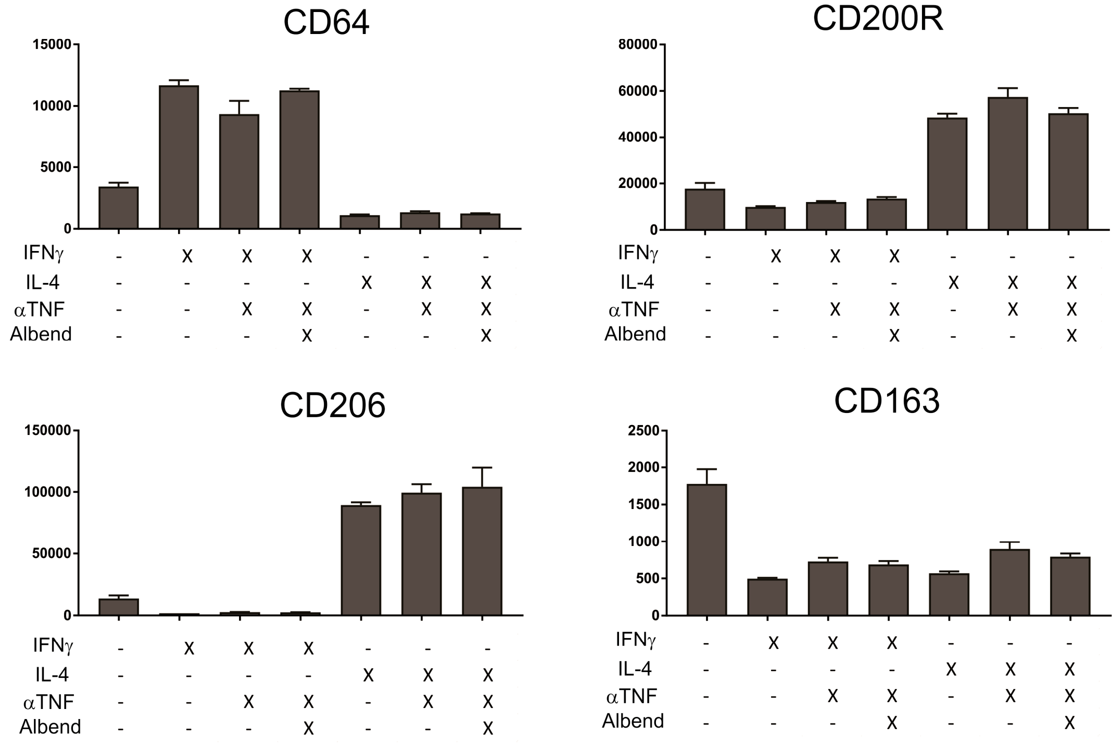

Fig S3

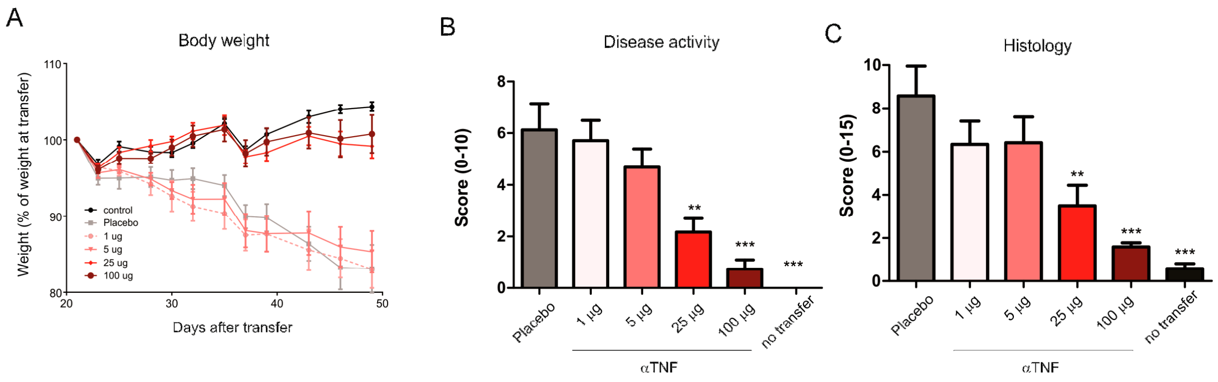
A
Body weight
Weight (% of weight at transfer)
Days after transfer
control
Placebo
1 ug
5 ug
25 ug
100 ug
B
Disease activity
Score (0-10)
Placebo
1 µg
5 µg
25 µg
100 µg
no transfer
αTNF
C
Histology
Score (0-15)
Placebo
1 µg
5 µg
25 µg
100 µg
no transfer
αTNF

Fig S4

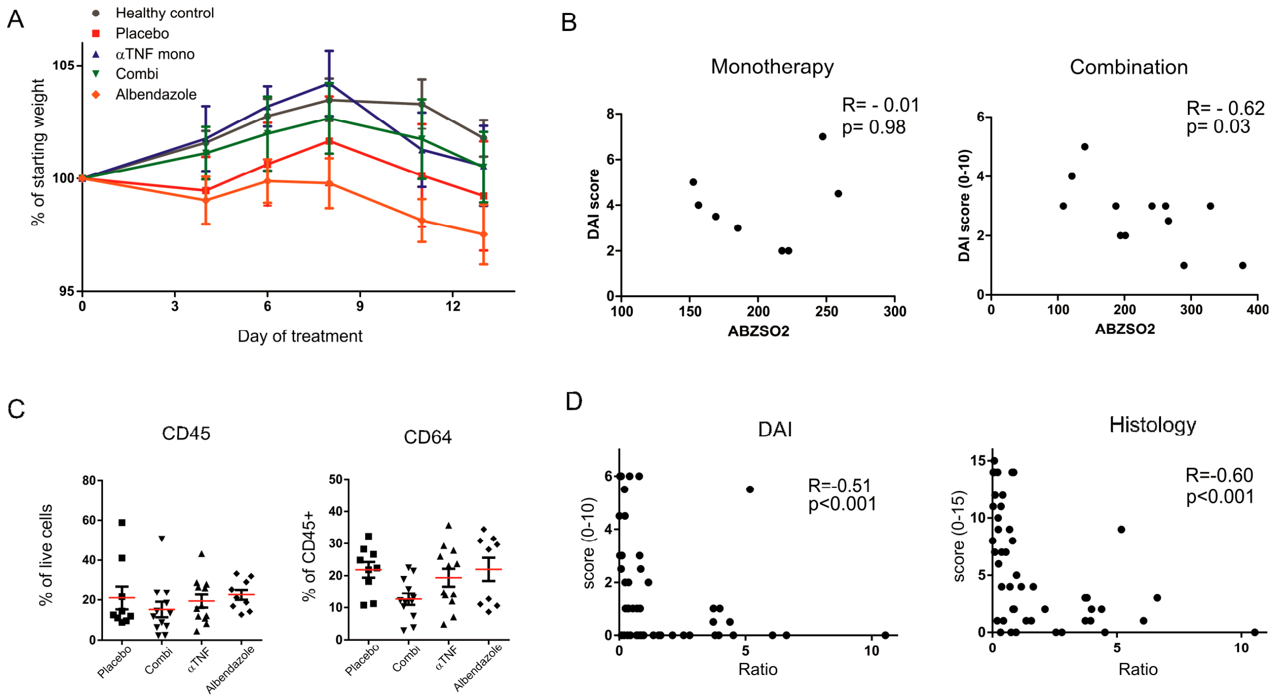
A
Healthy control
Placebo
αTNF mono
Combi
Albendazole
% of starting weight
Day of treatment
B
Monotherapy
R= - 0.01
p= 0.98
DAI score
ABZSO2
Combination
R= - 0.62
p= 0.03
DAI score (0-10)
ABZSO2
C
CD45
% of live cells
Placebo
Combi
αTNF
Albendazole
CD64
% of CD45+
Placebo
Combi
αTNF
Albendazole
D
DAI
R=-0.51
p<0.001
score (0-10)
Ratio
Histology
R=-0.60
p<0.001
score (0-15)
Ratio

Figure S5

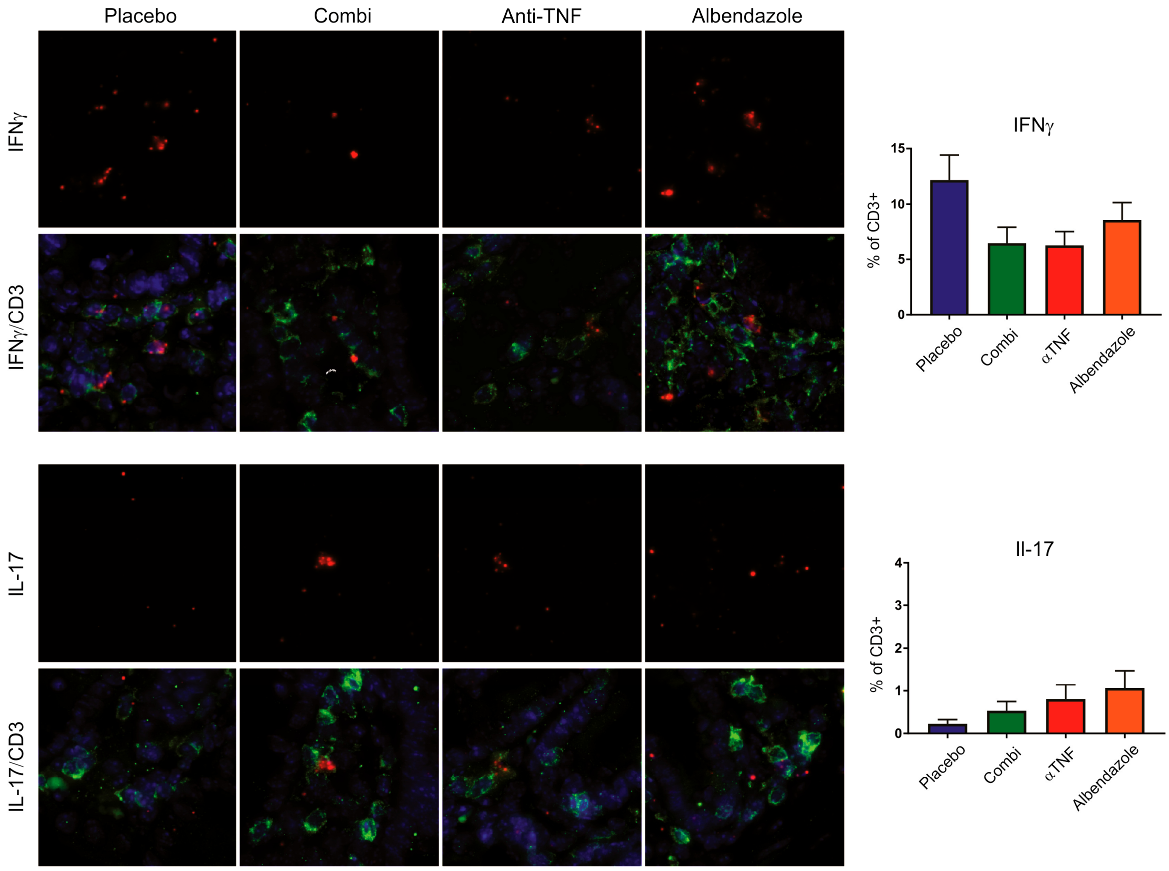
Placebo
Combi
Anti-TNF
Albendazole
IFNγ
IFNγ/CD3
IL-17
IL-17/CD3
IFNγ
15
10
5
0
% of CD3+
Placebo
Combi
αTNF
Albendazole
Il-17
4
3
2
1
0
% of CD3+
Placebo
Combi
αTNF
Albendazole

Supplementary table 1. Z-scores for all compounds positive in at least 1 concentration. Average Z-scores of duplicate plates shown.

Compound	Concentration			
	0.2	1	5	25
AMITRIPTYLINE HYDROCHLORIDE	-0.21013	0.322214	1.234258	2.346613
ADAPALENE	-0.5475	0.763549	1.910451	2.154424
DULOXETINE HYDROCHLORIDE	-0.74114	-0.15918	0.367986	2.106052
TRIAMTERENE	0.001886	-0.47369	2.061482	1.557107
PYRITHIONE ZINC	-0.64209	2.579317	-0.97384	-1.67921
BUDESONIDE	1.532911	2.253865	1.797801	3.668002
PYRIMETHAMINE	0.114936	0.176783	2.83361	2.663195
QUINACRINE HYDROCHLORIDE	-0.09225	0.193071	3.575685	2.594332
MEBENDAZOLE	-0.16922	1.56802	2.85073	2.414368
FENBENDAZOLE	-0.24569	2.541068	2.406356	1.21679
MYCOPHENOLIC ACID	1.800429	2.821401	2.65288	0.19034
CICLOPIROX OLAMINE	-0.10511	2.360678	2.706678	-0.74921
PACLITAXEL	2.6346	2.417066	2.500961	1.926552
ALBENDAZOLE	3.360264	2.825636	2.449469	1.306533
DOCETAXEL	3.166502	2.581564	2.918886	2.155076
NOSCAPINE HYDROCHLORIDE	-0.37264	0.242079	-0.10824	3.170651
DESIPRAMINE HYDROCHLORIDE	-0.27811	0.258515	0.265193	3.105973
ETICLOPRIDE HYDROCHLORIDE	-0.3262	0.084058	1.928713	2.952405
ESTRAMUSTINE	0.530788	0.613774	1.963704	2.904728
AMOXAPINE	-0.27811	0.410445	0.559109	2.823169
CLOMIPRAMINE HYDROCHLORIDE	-0.21177	-0.00932	-0.01678	2.720757
PROPAFENONE HYDROCHLORIDE	-0.85489	0.265568	0.763782	2.565126
PREDNISOLONE TEBUTATE	0.791886	1.440152	1.715264	2.529794
DESOXYCORTICOSTERONE ACETATE	-0.3597	1.094575	1.148207	2.430644
CHLORAMBUCIL	-0.36068	0.326826	0.786799	2.43049
MEXENEONE	0.308955	-0.03246	1.051118	2.327127
CHLORPROMAZINE	0.407455	0.026399	0.667394	2.301924
ALGESTONE ACETOPHENIDE	1.035327	0.96226	1.66756	2.255962
DOXIFLURIDINE	0.002169	0.226271	0.105737	2.248778
TRIMIPRAMINE MALEATE	0.004084	-0.29667	0.472779	2.244903
CARMOFUR	-0.52489	-0.31	0.554842	2.201726
PROMAZINE HYDROCHLORIDE	-0.19584	0.070909	0.163595	2.18804
OXFENDAZOLE	0.219738	0.293101	0.718779	2.143373
AZELASTINE HYDROCHLORIDE	0.07921	0.480723	-0.04314	2.138063
MEDRYSONE	1.117304	1.085268	1.369482	2.108899

Supplementary table 1 continued

Compound	Concentration			
	0.2	1	5	25
CARVEDILOL PHOSPHATE	0.146749	0.506456	0.524001	2.104324
ARTENIMOL	0.484976	0.243368	1.227673	2.092981
PROADIFEN HYDROCHLORIDE	-0.36808	-0.32316	0.599154	2.08231
MEBHYDROLIN NAPHTHALENESULFONATE	-0.0526	-0.02054	0.880855	2.05764
PIZOTYLINE MALATE	0.216726	0.043823	0.857083	2.01002
FLUOCINOLONE ACETONIDE	2.618878	1.797623	1.602268	1.976699
METHYLPREDNISOLONE SODIUM SUCCINATE	1.578721	1.644677	2.432871	1.872271
HYDROCORTISONE VALERATE	1.274497	1.515193	2.10057	1.805579
TRIFLURIDINE	0.600823	0.988232	2.168431	1.801951
BETAMETHASONE 17,21-DIPROPIONATE	1.859661	2.562602	1.945538	1.736798
DEXAMETHASONE ACETATE	0.846045	1.009726	2.687681	1.678094
AZACITIDINE	0.281067	0.45715	2.187213	1.540789
CLOBETASOL PROPIONATE	2.229596	1.857104	1.812835	1.526751
FLUMETHASONE	1.701586	2.332744	1.641873	1.463288
FLUOROMETHOLONE	3.010833	1.52352	1.491265	1.413105
FLUMETHAZONE PIVALATE	1.774908	2.026043	1.840133	1.397664
PREDNICARBATE	2.293694	0.657248	1.190237	1.357002
FLURANDRENOLIDE	2.137217	1.981236	1.546464	1.232692
MELENGESTROL ACETATE	1.125461	1.6228	2.630279	1.065071
BETAMETHASONE ACETATE	0.826679	1.445758	2.18329	1.040052
DESONIDE	2.051067	1.505478	1.708386	0.968862
FLUOCINONIDE	2.198473	1.911833	1.29463	0.921569
MYCOPHENOLATE MOFETIL	1.9427	2.362847	1.845194	0.857266
DEFEROXAMINE MESYLATE	0.599072	0.072822	2.913532	0.838002
CORTISONE ACETATE	1.689158	0.651001	2.239072	0.491429
FLORFENICOL	0.20162	-0.11717	2.032589	0.241095
IRINOTECAN HYDROCHLORIDE	1.409696	2.401522	1.672785	-0.0398
NONOXYNOL-9	0.095552	1.647247	2.131446	-0.26221
HYDROXYPROGESTERONE CAPROATE	0.945514	1.048604	2.045631	-0.34291
ASTEMIZOLE	0.066807	0.858036	2.237174	-1.36142
MITOMYCIN	1.069483	2.701959	0.330301	-1.39736
NITROXOLINE	0.018503	0.005595	2.509718	-1.5807
DAUNORUBICIN	1.659351	2.056357	-1.75633	-1.64623
METERGOLINE	0.26819	1.677951	2.860256	-1.72649
RALOXIFENE HYDROCHLORIDE	0.419032	0.547076	2.380362	-1.77627

Supplementary table 1 continued

Compound	Concentration			
	0.2	1	5	25
CEPHARANTHINE	2.299124	1.718706	1.338149	-1.82841
ABAMECTIN (avermectin B1a shown)	0.503151	0.531683	2.270143	-1.84179
TENIPOSIDE	2.136213	1.770871	0.109522	-1.87601
ANCITABINE HYDROCHLORIDE	1.76973	2.276456	-1.22969	-1.88075
THIMEROSAL	1.339509	2.13363	-1.97186	-1.93679
CHLOROACETOXYQUINOLINE	-0.11704	0.127474	2.57679	-1.96745
BECLOMETHASONE DIPROPIONATE	1.953924	2.360678	1.735954	3.57427
BETAMETHASONE SODIUM PHOSPHATE	1.84469	2.221734	2.135142	1.652565
DEXAMETHASONE SODIUM PHOSPHATE	2.12349	1.469737	2.096754	1.594916
MOMETASONE FUROATE	2.147394	2.016002	1.769189	1.593796
METHOTREXATE(+/-)	2.718708	1.937627	2.307606	1.556972
FLUBENDAZOLE	1.180537	3.237184	3.032643	1.398432
BETAMETHASONE VALERATE	2.056343	2.338086	1.643522	1.348377
THIOGUANINE	0.041558	2.371629	2.753582	1.341025
BROXALDINE	0.128261	2.07337	2.026002	1.317227
FLUDROCORTISONE ACETATE	1.986691	2.079341	2.239746	1.109433
EPIRUBICIN HYDROCHLORIDE	2.257973	2.086557	1.630717	-1.7861
DEXAMETHASONE	2.400269	1.760937	2.502049	2.049619
OXIBENDAZOLE	3.092254	3.307073	3.04458	1.986365
HYDROCORTISONE	2.355853	2.010847	2.708082	1.473254
BETAMETHASONE	2.438484	2.835705	2.210531	1.465903
FLUTICASONE PROPIONATE	2.771427	2.448814	2.032982	1.142261
DEFLAZACORT	2.153297	2.313795	2.334981	0.929497
AMSACRINE	2.711551	2.513484	2.428708	-1.30019
AMINOPTERIN	2.486678	3.072685	2.89586	2.293416
ETOPOSIDE	2.255381	2.393979	2.582695	2.289404
FLOXURIDINE	4.107263	4.112206	3.627304	2.240838
PEMETREXED	2.004285	3.213089	2.381534	2.007305

Supplementary table 2. Comparison of initial screen results and validation in a secondary laboratory. For screen '+' indicates Z-score>2, for validation experiment '+' indicates significantly enhanced induction compared to IFX alone (n=5/condition).

Compound	Concentration (μM)							
	0.2		1		2.5/5		25	
	Screen	Validation	Screen	Validation	Screen	Validation	Screen	Validation
PYRITHIONE ZINC	-	-	+	+	-	-	-	-
CICLOPIROX OLAMINE	+	-	-	-	-	-	-	-
MEBENDAZOLE	-	+	-	+	+	+	+	+
PYRIMETHAMINE	-	-	-	-	+	-	+	+
FENBENDAZOLE	-	-	+	+	+	+	-	-
ALBENDAZOLE	+	+	+	+	+	+	-	+
QUINACRINE HYDROCHLORIDE	-	-	-	+	-	+	+	-
PACLITAXEL	+	+	+	+	+	+	+	+
DOCETAXEL	+	+	+	+	+	-	+	-
MYCOPHENOLIC ACID	+	+	+	+	+	+	-	+
ADAPALENE	-	-	-	-	+	+	+	-
AMITRIPTYLINE HYDROCHLORIDE	-	-	-	-	-	-	+	-
DULOXETINE HYDROCHLORIDE	-	-	-	-	-	-	+	-
BUDESONIDE	+	-	+	+	-	+	+	+
TRIAMTERENE	-	-	-	-	+	-	-	+
